# Etiology of right ventricular restrictive physiology early after repair of tetralogy of Fallot in pediatric patients

**DOI:** 10.1186/s13019-019-0909-8

**Published:** 2019-05-02

**Authors:** Bhushan Sandeep, Xin Huang, Fan Xu, Pengxiao Su, Ting Wang, Xiaoke Sun

**Affiliations:** 1 Department of General Surgery, Honghui Hospital, Xi’an Jiaotong University, Xi’an, 710000 Shanxi China; 2 Chengdu medical college, Jinniu district, Rondu avenue, Tianzhu road no 611, Chengdu, 610500 China

**Keywords:** Tetralogy of Fallot (TOF), Right ventricular restrictive physiology (RVRP), End-diastolic forward flow (EDFF), Cardiopulmonary bypass (CPB)

## Abstract

**Background:**

Right ventricular restrictive physiology (RVRP) is a common finding after repair of Tetralogy of Fallot (TOF). The characteristic feature of RVRP is the presence of a direct end-diastolic flow (EDFF) during atrial contraction in the main pulmonary artery. This end-diastolic forward flow is caused by increased right ventricular end-diastolic pressure due to right ventricular myocardial stiffness and decreased right ventricular compliance.

**Objective:**

Our main objective is to found out the etiology of RVRP in pediatrics patients who underwent for complete repair of Tetralogy of Fallot (TOF).

**Methods:**

A total of 50 TOF patients have registered for this study in our hospital from January 2017 to September 2018. The patients were divided in two groups, group A with restrictive physiology and group B without restrictive physiology. The patients selected for this study includes TOF patients, TOF patients with atrial septal defect (ASD), and TOF patients with patent ductus arteriosus (PDA). Ventricular hypertrophy and right heart enlargement were evaluated by electrocardiogram and echocardiography. The other parameters we used to compare between these two groups were sex, age, weight, cardio pulmonary bypass (CPB) time, aortic cross clamping time, transannular patch, SP0_2_, RV/LV pressure, ventricular hypertrophy, right heart (RH) enlargement, tricuspid annular plane systolic excursion (TAPSE), pulmonary artery systolic pressure (PASP), TAPSE/PASP ratio, pulmonary annular diameter, intubation time, PICU stay and hematocrit (HCT).

**Results:**

RVRP was identified in 28 patients (58%). Lower SP0_2_ (mean: 84.3 ± 7.9%) with *p*-value 0.015, transannular patch repair (*n* = 22, 78.5%) with p-value< 0.001, longer cardiopulmonary bypass (CPB) time (mean: 117.6 ± 23 min) with p-value< 0.001, longer aortic cross clamping time (mean: 91.4 ± 20.26 min) with p-value< 0.001, lower TAPSE, lower PASP,lower TAPSE/PASP ratio and presence of hypertrophy (p-value < 0.001) were identified as etiology for restrictive physiology. It was also found that 77% TOF patients with ASD have a higher risk of RVRP in our study.

**Conclusions:**

In TOF patient’s etiology for right ventricular restrictive physiology are associated with lower SP0_2,_ transannular patch repair, longer CPB and longer aortic cross clamping time, hypertrophy, lower TAPSE, lower PASP and lower TAPSE/PASP ratio.

## Introduction

TOF is the most common cyanotic congenital heart disease that represents 55–70% of cyanotic congenital heart disease. Total correction at an early age is the main surgical management in TOF patients. After total repair of TOF, many patients suffer a slow postoperative recovery, with an evidence of a raised central venous pressure, fluid retention, pleural effusion, a low cardiac output, ascites, right ventricular myocardial delayed enhancement and diastolic dysfunction [1, 2]. In the absence of significant residual ventricular septal defect (VSD) and obstructive lesions etc., it is very common that such patients have RVRP. The incidence of RVRP varies from 50 to 70% [3]. On the basis of echocardiography, RVRP is defined as the presence of end –diastolic forward flow (EDFF) into the main pulmonary artery. This presence of end –diastolic forward flow in the pulmonary arteries, if persistent throughout the respiratory cycle has been regarded as hallmark of right ventricular restriction [4]. The pathophysiological cause of right ventricular restriction is an increase in right ventricular end diastolic pressure due to increased myocardial stiffness and decreased compliance of the right ventricle. This restriction of the right ventricle results in a high systemic venous pressure which is often associated with prolonged pleural effusion and a reduced cardiac output that results in a prolonged stay in the intensive care unit (ICU) [5]. The strategy used for restrictive patients to improve early recovery are optimize cardio protection, pulmonary monocusp, leave ASD shunt, aggressive drainage of fluid in thoracic and abdominal cavity, maintain high central venous pressure (CVP) and extubation of patient as early as possible. As the postoperative management of restrictive TOF patients are different from the nonrestrictive TOF patients the prediction of etiology for RVRP can help to improve the clinical management and early recovery of restrictive TOF patients. Previous studies have shown that RVRP is a transient phenomenon that resolves in two weeks but the etiology of restrictive physiology is not clear. Some studies have shown that the transannular patch is one of the risk factor for restrictive physiology but do not mention the other factors responsible for this. We therefore conducted a retrospective study comparing restrictive patients with non restrictive patients who underwent for total correction of TOF using various parameters such as age, weight, SP0_2_, HCT, ventricular hypertrophy, right heart enlargement, transannular patch repair, CPB time, aortic cross clamping time, RV/LV pressure ratio,TAPSE, PASP, TAPSE/PASP ratio and an attempt was made to find the etiology of RVRP. Therefore, the purpose of this study is to characterize the clinical, pre and post surgical and echocardioagraphic features of those patients with restriction and without restriction to determine which variables could be associated with RVRP.

## Patients and methods

Study population: The protocol was approved by the Honghui Hospital, Xi’an Jiaotong University Clinical Research Ethics Committee, and parents of all subjects provided informed consent. We retrospectively studied 50 consecutive patients admitted in our hospital for total repair of TOF between the periods of January 2017 to September 2018. Patients with a documented primary diagnosis of TOF were identified by review of clinic schedules and approached for transthorasic and transesophageal echocardiography. The patients were divided into two groups, group A (*n* = 28) having restrictive physiology and group B (*n* = 22) without restrictive physiology. We assessed all these 50 patients prospectively and we measured their age, weight, SP0_2_ at the time of admission and HCT by blood routine test. For right ventricular evaluation we examined the 12 lead electrocardiograph and echocardiography for finding right ventricular hypertrophy and right heart enlargement. Right ventricle/Left ventricle (RV/LV) pressure ratio was calculated by insertion of needle during surgery. In our study we selected only those patients whose diagnosis was TOF, TOF with ASD, TOF with PDA and one stage complete repair. We excluded TOF patients associated with other cardiac malformation such as muscular VSD, unilateral PA, valve regurgitation and previous palliation. For the confirmed diagnosis of TOF transthoracic and transesophageal echocardiography and an X-ray examination were performed. Total correction of TOF was performed during surgery using standard CPB and by aortic cross clamping under moderate hypothermia. The VSD and ASD were repaired during surgery using either patient’s pericardium or Dacron patch, PDA (*n* = 6) was ligated. The sutures used for closing VSD were polypropylene. Hypertrophied tissues of right ventricle and fibrotic tissues of right ventricular outflow tract have been removed during surgery. Augmentation of main pulmonary artery and transannular patching (*n* = 28) were done during aortic cross clamping using patient’s pericardium or using a bovine pericardium patch if needed. Type of repair, CPB time, aortic cross clamping time were recorded during time of surgery. RVRP was identified just after surgery and in pediatric intensive care unit (PICU) using transthorasic echocardiography with respiratory monitoring by the presence of end-diastolic forward flow in main pulmonary artery. We also observed the total duration of intubation and the number of times the patient was intubated and the total duration of the stay in the PICU.

Echocardiography: Transthorasic echocardiography was performed for each patient with commercially available echocardioagraphic equipment with simultaneous recording of electrocardiographic and respiratory waveforms. Complete transesophageal echocardiography was performed for each patient just before surgery in surgery room with respiratory monitoring. The transesophageal echocardioagraphic probe was placed according to the usual techniques and the examinations were performed in a standard manner with echocardioagraphic experts [6].

RV hypertrophy is echocardiographically defined as a ventricular wall thickness more than 5 mm at end-diastole [5]. We used M-mode and 2-dimensional echocardiography for measuring RV wall thickness to find out the hypertrophy of right ventricle [7]. For measuring hypertrophy we divided patients in two groups having RV wall thickness 5 mm and greater than 5 mm. RV free wall assessment is best performed from the apical and subcostal 4-chamber views. Increased RV free wall thickness or hypertrophy as measured by echocardiography has been well validated and usually indicates pressure overload [8]. Guidelines from the American Society of Echocardiography recommend the use of the subcostal 4-chamber view for measurements of RV free wall thickness, because it has demonstrated higher reproducibility [9]. For right heart enlargement we use two-dimensional echocardiography, with the apical four chambered view which enables accurate visualization of the right atrium and right ventricle in all patients.. Tricuspid annular plane systolic excursion (TAPSE), Pulmonary artery systolic pressure (PASP) and TAPSE/PASP ratio were calculated with the help of echocardiography.

Clinical assessment: An extensive clinical evaluation was carried out for each patient after being admitted to our hospital considering a particular attention to his signs and symptoms. Before the surgery a routine blood test and a pre-surgical discussion with experts were performed.

Statistical analysis: Quantitative variables were expressed as means ± standard deviation and compared by using Student t test. Means and ranges are given for continuous demographic variables. We expressed categorical variables as number or percentages and made comparison using χ2 analyses or Fisher exact test. A bidirectional hypothesis was applied and significance was considered with *p* < 0.05. The statistical software SPSS version 19.0 was used for all statistical analysis.

## Results

The demographic data and clinical outcome are presented in Table 1. Restrictive physiology was found in 28 patients (58%) out of 50. Both groups were compared in terms of age, sex, weight, HCT, SP0_2_, transannular patch repair, ventricular hypertrophy, right heart enlargement, CPB time, aortic cross clamping time, pulmonary annular diameter, TAPSE, PASP, TAPSE/PASP ration, RV/LV pressure gradient and TOF patients with ASD and PDA.

**Table 1:** variables of risk factor in restrictive and non restrictive group

Variables	Group A	Group B	*P* value
No of patients	28	22	
Sex(M/F)	20/8	12/10	0.240
Age (months)	30.71 ± 28.52	20 ± 10.76	0.094
Weight (kg)	10.93 ± 4.74	10.10 ± 2.31	0.455
CPB time(minute)	131 ± 20.7	100.2 ± 12.1	< 0.001*
Aortic cross clamping time (minute)	102.89 ± 17.14	76.40 ± 13.74	< 0.001*
Transannular patch	22/28	6/22	< 0.001*
Spo_2_ (%)	79 ± 8.4	87 ± 6	0.015*
RV/LV pressure	67.95 ± 5.69	63.31 ± 5.66	0.009*
Ventricular hypertrophy			
1.5 mm	7	16	< 0.001*
2. > 5 mm	21	6	< 0.001*
RH enlargement	15	4	< 0.001*
TAPSE mm Hg	13.41 ± 1.35	16.70 ± 1.51	< 0.001*
PASP mm Hg	49.15 ± 6.75	33.54 ± 5.48	< 0.001*
TAPSE/PASP	0.27 ± 0.05	0.51 ± 0.15	< 0.001*
TOF pts.	20	17	
TOF pts. with PDA	3	3	
TOF pts. with ASD	5	2	
Pulmonary annular diameter(mm)	10.22 ± 2.1	12.31 ± 2.31	0.115
Intubation time(minute)	382.35 ± 64.52	219.36 ± 44.28	< 0.001*
PICU stay(days)	8.92 ± 1.24	4.15 ± 1.18	< 0.001*
HTC (l/l)	0.45 ± 0.05	0.39 ± 0.02	< 0.001*

*M* male, *F* female, *TOF* Tetralogy of Fallot, *PDA* Patent ductus artriosus, *ASD* Atrium septal defect, *CPB* Cardio pulmonary bypass, *TAP* Trans annular patch, *RV* Right ventricle, *LV* left ventricle, *RVRP* Right ventricle restrictive physiology, *RH enlargement* Right heart enlargement, *TAPSE* Tricuspid annular plane systolic excursion, *PASP* Pulmonary artery systolic pressure

**Fig. 1:**
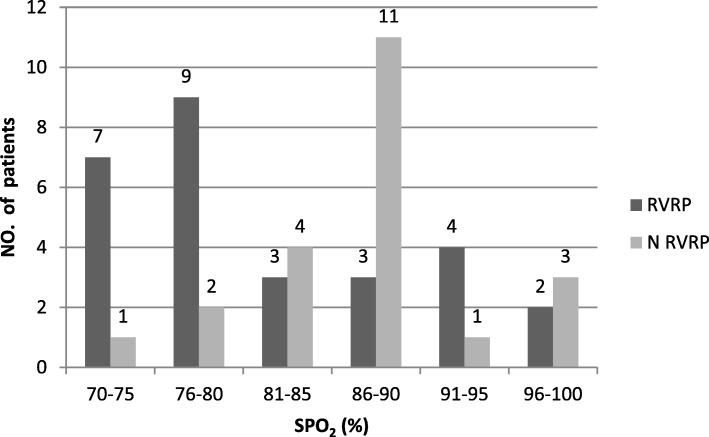
Comparison of RVRP and N RVRP patients on SP0_2._ RVRP (Right ventricular restrictive physiology), NRVRP (No right ventricular restrictive physiology)

The major risk factors for restrictive physiology identified in our study were longer CPB time, longer aortic cross clamping time, transannular patch repair, low SP0_2,_ RV/LV pressure, ventricular hypertrophy, lower TAPSE, lower PASP, lower TAPSE/PASP ratio, and TOF patients with ASD. In our study males predominates females (64%). In restrictive group 71.4% males had restrictive physiology. In our study the mean age of restrictive patients was higher than non restrictive patients but the *p*-value was not significant. Sex, age and weight of patients did not appear as an etiology for RVRP. We measured the Hematocrit (HCT) from blood routine test. The HCT value for restrictive patients were higher than non restrictive patients with a *p*-value< 0.001. Transannular patch was used in 28(56%) patients, and a major statistical difference was found for the incidence of RVRP. In group A: 78.5% patients had RVRP who used transannular patch while in group B only 27.2% patients identified with RVRP who did not received transannular patch repair, therefore transannular patch repair shows a higher risk factor for RVRP (78.5%) with a p-value < 0.001. According to electrocardiographic and echocardioagraphic measurements we identified 27 patients having RV wall thickness > 5 mm and 23 patients having wall thickness ≤ 5 mm. The statistical difference between these hypertrophied patients having restrictive physiology were 77.7 and 30.4% respectively. We identified hypertrophied patients having wall thickness > 5 mm are on greater risk for RVRP with a *p*-value< 0.001. From two-dimensional echocardiography with the apical four chambered view we identified 19 patients having right heart enlargement in which 15 patients had RVRP (p-value< 0.001). The mean pulmonary diameter of restrictive patents was less than non restrictive patients but the p-value was not significant so pulmonary annular diameter did not appeared as a risk factor. In our study 37 patients have been diagnosed as TOF without ASD and PDA, among these 37 patients 20 patients had RVRP. Out of total 6 TOF patients with PDA only 3 showed RVRP and 3 were without RVRP, so the incident rate of RVRP was 50% in our study population. In our study TOF patients with ASD (*n* = 7) showed a high incidence (71%) of RVRP. In total 7 patients 5 showed RVRP. In our study the SP0_2_ value less than 80% showed a high risk for RVRP because we have 19 patients whose SP0_2_ were between 70 and 80% and out of these 19 patients; 16 patients showed RVRP. As the SP0_2_ value goes higher the incidence of RVRP decreased. In Fig. 1, we can see the variation of SP0_2_ with RVRP.

**Fig. 2:**
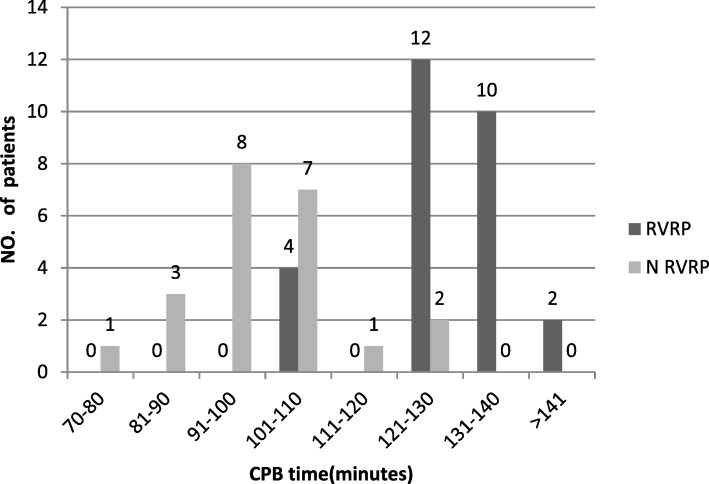
Comparison of RVRP and N RVRP patients on CPB time

Cardio pulmonary bypass (CPB) time: At the time of surgery we measured the CPB time for all patients ranging from 71 to 205 min. We found that when the CPB time was less than 100 min there was no case of restriction but when the CPB times increases by 100 min the risk of restriction also increases gradually. The RVRP rate was 85% when the CPB time was between 121 and 130 min and it was 100% when the duration of the CPB was more than 131 min. In our study we have two patients whose CPB time was exceptionally longer 181 and 205 min respectively and it was found that both had RVRP. In Fig. 2 we can see the variation between CPB time and patients having RVRP and patients without RVRP.

**Fig. 3:**
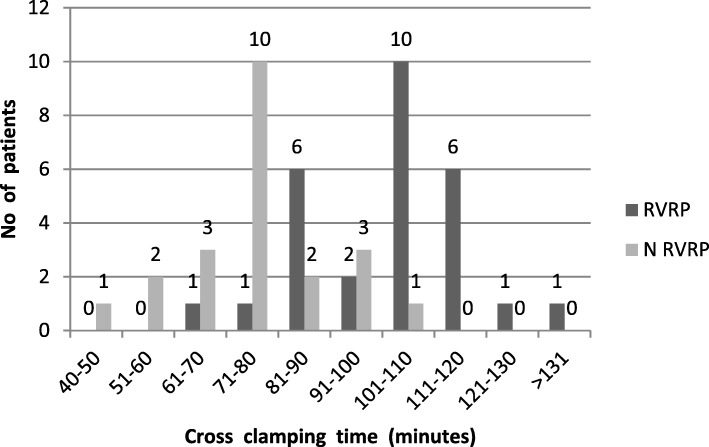
Comparison of RVRP and NRVRP patients on Cross clamping time

Aortic cross clamping time: We measured aortic cross clamping time during surgery. The risk of RVRP was higher (94.7%) in patients whose aortic cross clamping time was more than 100 min, in total 19 patients whose aortic cross clamping time was more than 100 min 18 have RVRP. Rest 31 patients whose aortic cross clamping time was less than 100 min only 18 have RVRP. In Fig. 3 the distributions of aortic cross clamping time and patients with restrictive physiology and without restrictive physiology is shown.

The higher RV/LV pressure ratio for restrictive group with a *p*-value 0.009 shows a risk factor for RVRP in our study. On basis of echocardiography results we found that TAPSE, PAPSE and TAPSE/PASP ratio was lower in restrictive patients. The p-value for TAPSE, PASP and TAPSE/PASP ratio was < 0.001 in restrictive patients After comparing both of these groups on the basis of echocardiography parameters lower TAPSE, PASP and TAPSE/PASP ratio came out as a strongest predictor for risk factor for RVPA. During PICU stay we measured the intubation time for every patient, the mean intubation time for restrictive group was almost double than non restrictive group. We also calculated the total time duration of PICU stay, the mean time duration of PICU stay for restrictive group was 8.21 ± 2.11 days and for non restrictive group it was 4.20 ± 1.91 days, so for the restrictive group the time duration of PICU stay was almost double from non restrictive group with a significant *p*-value.

## Discussion

This is the first study of our knowledge that focuses mainly on the etiology of RVRP not on the mechanism of RVRP in pediatric patients since most of the previous studies have been performed in adolescent or adult patients. RVRP exists in a significant number of patients who underwent for total surgical repair of TOF and have been evaluated for diastolic dysfunction (end-diastolic forward flow). RVRP is defined by the presence of end-diastolic forward flow (EDFF) during atrial contraction into the main pulmonary artery. The presence of end-diastolic forward flow in the pulmonary arteries, if persistent throughout the respiratory cycle, has been regarded as hallmark of restriction of the right ventricle, when assessed through transthorasic and transesophageal echocardiography. This right ventricular restriction is caused by increased right ventricular end –diastolic pressure due to increased myocardial stiffness and decreased right ventricular compliance. The reduction in right ventricular diastolic compliance is reflected as antegrade forward flow through the superior vena cava. Due to this restriction the right ventricle acts as a passive conduit between the systemic venous inflow and pulmonary artery inflow during atrial systole [10].

We found that RVRP occurs when patients have lower SP0_2_(< 80%), heigher HCT value, transannular patch repair, longer CPB time (> 110 min), longer aortic cross clamping time(> 100 min), lower TAPSE, PASP and TAPSE/PASP ratio and presence of ventricular hypertrophy. In our study male patients predominates in both groups. SP0_2_ is determined by cardiac output and the arterial venous oxygen difference, age, sex, muscle mass, genetic endowment, lung function, and efficacy of exercise. Restrictive patients in this study had a lower SP0_2_ than non restrictive patients. Any reduction in SP0_2_ coincides with a reduction in inventilation capacity. For the restrictive group the mean age was higher than non restrictive group. The sex, age and weight of patients did not show any close relationship as a risk factor of RVRP. The reason behind this is we did not included adult patients in our study so we can’t make a comparison for age and weight [11]. Some previous studies say that greater the age of patient higher the possibility of RVRP but other researchers contradict it [12]. In our study and also in some previous studies it has been shown that the repair of transannular patches is an important risk factor for RVRP. We had a total of 6 patients who had TOF with PDA and of these 6 only 3 showed RVRP and 3 without RVRP so the incidence of RVRP was 50% in our study population but as TOF patients with PDA are very few in number we can not conclude that the TOF with PDA is a risk factor for RVRP. In the current study, indexing TAPSE to PASP did significantly strengthen the association between restrictive and non restrictive patients and RV function, suggesting that this simple echocardiography measure can supports further understanding into the severity of physiological evaluation in TOF patients.

Most of the previous articles discuss the mechanism of RVRP the clinical outcome and the echocardiographic characteristics. Some papers deals with the association of RVRP with the myocardial injury and oxidative stress, influence of restrictive physiology on LV diastolic function [12]. It is very rare to find in previous studies that deal with the etiology of RVRP. Some previous studies found that RVRP appeared more predominant after transannular patch repair [13, 14]. Norgard et al. also found, by multiple logistic regression analysis that right ventricular restrictive physiology is more likely related to the anatomic substrate requiring a transannular patch repair [15]. Clark AL and their colleagues also suggest the same that transannular patch repair is main risk factor for restrictive physiology [16]. In our study it was also found that restrictive physiology is correlated with the repair of transannular patches (76.5%).The reason behind this is the incidence of RV dilatation is higher in patients who had a transannular patch and this makes the right ventricle more susceptible to gradual dilatation as observed by Yetman and colleagues [3]. Mulla and colleagues reported that restrictive physiology is limited by RV dysfunction not by use of a transannular patch [3]. However this was contradicted by Mahle and colleagues [17]. Mulla and colleagues also demonstrated that there is no any association between age of the patient at total correction of TOF and restrictive physiology [12], but in our study we found that restrictive group had higher mean age than non restrictive group but the *p*-value was not significant. In most of the previous studies the patients are adolescents or adults and their age is variable but in our study the age of our patients ranges between 5 months to 10 years and we did not include any adult patient in our study so we can not make any evaluation and conclusion whether the patient’s age and weight is a risk factor. [18]. Rajiv R Chaturvedi and his colleagues described that acute right ventricular restrictive physiology is associated with greater intraoperative myocardial injury and postoperative oxidative stress with severe iron loading of transferrin, the cause of myocardial injury can be happened due to longer cardiopulmonary bypass(CPB) time and longer duration of aortic cross clamping time [19]. In our study we also found that patients who have a longer duration of PCB and cross-clamping and more likely to show restrictive physiology. Therefore by minimizing CPB time and cross-clamping we can reduce myocardial injury and reduce the chances of RVRP. Peter Munkhammar and his colleagues described a strong association between the right ventricular restrictive physiology detected on MRI and fibrosis of the RVOT in children after repair of TOF, the link may be that the fibrosis decreases the RV compliance [20]. In this case, RV with low compliance at atrial systole blood will pumped against a stiff right ventricle, resulting in forward pulmonary flow in ventricular diastole. In our study we also found a close relationship of RVOT fibrosis with RVRP but our technique used to detect fibrosis is echocardiography and we only have 2 patients who undergo an MRI exam before surgery so we exclude RVOT fibrosis of our study.

### Limitations

Our study has been performed on a relatively smaller number of pediatric patients and we did not include adult patients in this study so we cannot say if age and weight of the patient has any etiological relationship with RVRP. However in our study transesophageal echocardiography was very useful to find out the ventricular enlargement and ventricular hypertrophy. But the best modality to find out the degree of ventricular enlargement, degree of ventricular hypertrophy and degree of fibrosis at RVOT is MRI and we have only 2 patients in our study undergo for MRI examination. It is also possible that our parameters to find out the risk factors may not always be sufficient and specific and there always be exceptions so our results are suboptimal.

## Conclusions

This is the first study of our knowledge conducted in pediatric TOF patients focousing on etiology of RVRP. This study suggests that etiology of RVRP is associated with low SP0_2,_ HCT, ventricular hypertrophy, right ventricular enlargement, transannular patch repair, and longer duration of cardiopulmonary bypass and aortic cross clamping, low TAPSE, low PASP, low TAPSE/PASP ratio and after knowing the etiology we can manage the post surgical in better ways.. This study also suggest that avoiding transannular patch and minimizing CPB time and cross clamping to prevent myocardial injury can improve incidence of RVRP.However, further study in a larger population is required to confirm these findings and clarify detailed characteristics of right ventricular restrictive physiology in repaired TOF patients.

## Abbreviations

ASD: Atrial septal defect
CPB: Cardio pulmonary bypass
CVP: Central venous pressureEDEF: End diastolic forward flow
HCT: Hematocrit
LV: Left ventricle
PASP: Pulmonary artery systolic pressure
PDA: Patent ductus arteriosus
PICU: Pediatric intensive care unit
RH: Right heart
RV: Right ventricle
RVRP: Right ventricle restrictive physiology
TAPSE: Tricuspid annular plane systolic excursion
TOF: Tetralogy of Fallot
